# Discovery of a new *Theileria* sp. closely related to *Theileria annulata* in cattle from Sri Lanka

**DOI:** 10.1038/s41598-019-52512-y

**Published:** 2019-11-06

**Authors:** Thillaiampalam Sivakumar, Shiori Fujita, Bumduuren Tuvshintulga, Hemal Kothalawala, Seekkuge Susil Priyantha Silva, Naoaki Yokoyama

**Affiliations:** 1 National Research Center for Protozoan Diseases, Obihiro University of Agriculture and Veterinary Medicine, Hokkaido, Japan; 2 Veterinary Research Institute, Peradeniya, Sri Lanka; 3 OIE reference laboratory for bovine babesiosis and equine piroplasmosis, National Research Center for Protozoan Diseases, Obihiro University of Agriculture and Veterinary Medicine, Hokkaido, Japan

**Keywords:** Speciation, Parasite evolution

## Abstract

*Theileria annulata* is a haemoprotozoan parasite that causes a cancer-like illness known as tropical theileriosis in cattle. In the course of analyzing the genetic diversity of *T*. *annulata* in Sri Lanka, we observed that merozoite-piroplasm surface antigen (*tams1*) and surface protein (*tasp*)-like gene sequences obtained from bovine blood DNA samples, which were PCR-positive for *T*. *annulata*, were conserved but shared low identity with *T*. *annulata* GenBank sequences. Moreover, the 18S rRNA sequences from the Sri Lankan samples contained ten unique single-nucleotide polymorphisms compared with all known *T*. *annulata* sequences. The cytochrome b (*cob*) gene sequences isolated from the Sri Lankan samples were highly conserved and shared low identity scores with similarly conserved *T*. *annulata* sequences from GenBank. Phylogenetic analysis showed that the Sri Lankan *tams1*-like, *tasp*-like, 18S rRNA, and *cob* sequences clustered together and formed sister clades to the common ancestors of all known *T*. *annulata* and *Theileria lestoquardi* sequences. These findings demonstrated that the Sri Lankan cattle were not infected with *T*. *annulata* but with a new *Theileria* sp. (designated as *Theileria* sp. Yokoyama) closely related to *T*. *annulata*.

## Introduction

The *Theileria* genus consists of tick-borne haemoprotozoan parasites of ruminants^1^. Among them, *Theileria annulata* infects cattle and buffalo, and causes a lymphoproliferative disease known as tropical theileriosis^2^. The disease is usually characterised by enlarged lymph nodes, fever, increased pulse and respiratory rates, swollen eyelids, profuse lachrymation, anaemia, jaundice, and sometimes death^3,4^. Therefore, tropical theileriosis is considered an economically significant disease in endemic countries^5–7^. Long-term survival of *T*. *annulata* in host animals is facilitated by the protozoan’s genetic diversity, which helps the parasite escape the host’s immune response^8,9^. Therefore, *T*. *annulata*’s genetic diversity has been analysed in several endemic countries based on genes that encode surface proteins, such as the *Theileria annulata* merozoite-piroplasm surface antigen (*tams1*) and *Theileria annulata* surface protein (*tasp*) genes^10–13^. The *tams1* and *tasp* genes have been demonstrated to be highly diverse in most endemic countries investigated to date.

Sri Lanka is a tropical country, and previous PCR-based studies found that infection with *T. annulata* is common among the country’s cattle populations^14,15^. However, the genetic diversity of *T*. *annulata* remains to be investigated in Sri Lanka. Therefore, in the present study, we analysed the *tams1* and *tasp* gene sequences in DNA samples from Sri Lankan bovine blood, which were PCR-positive for *T*. *annulata*. As the findings suggested that the genetic backgrounds of Sri Lankan isolates might be different from those of *T*. *annulata*, further analyses were conducted using the 18S rRNA and cytochrome b (*cob*) gene sequences.

## Results

**Figure 1:**
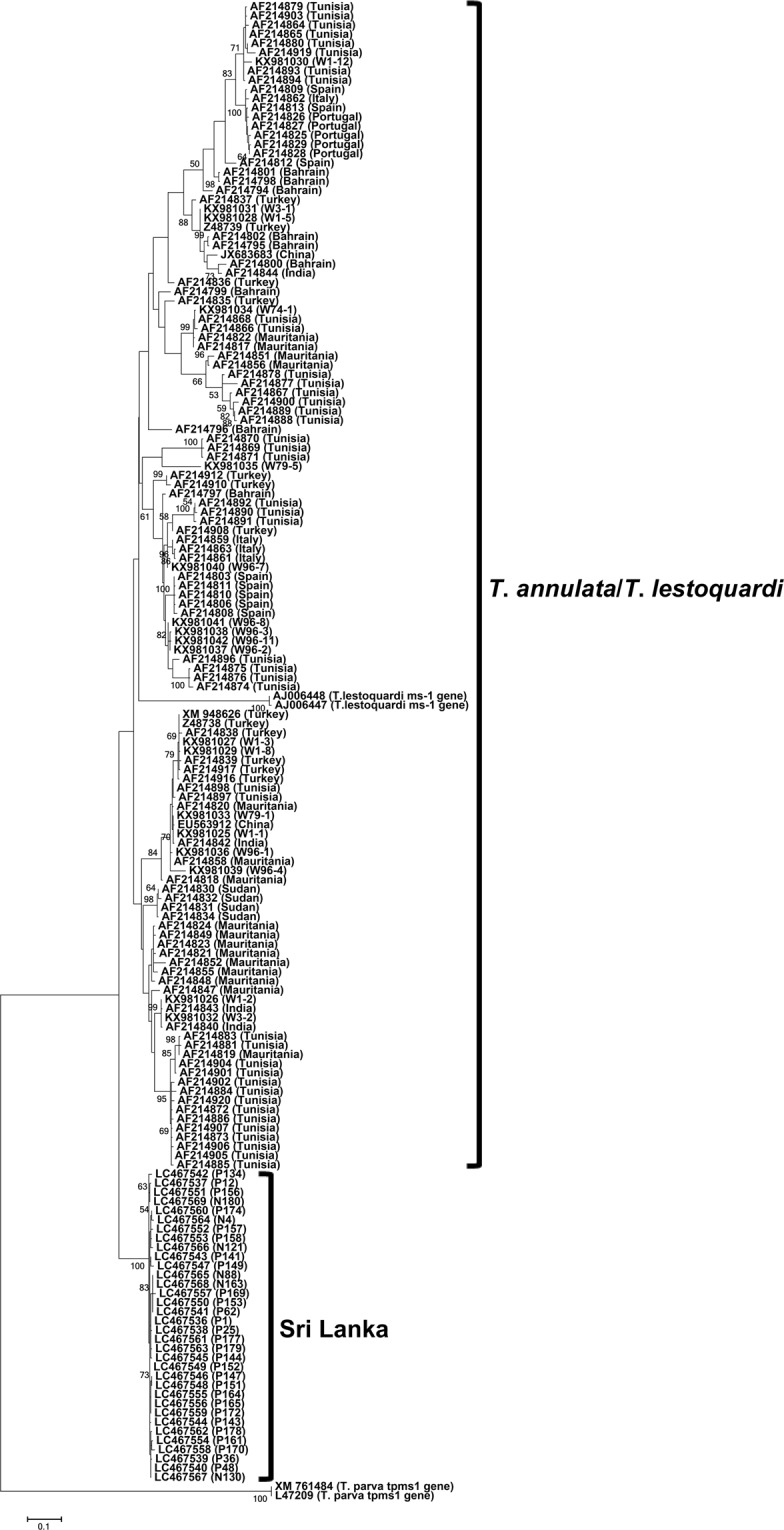
Phylogeny of the *tams1* gene. A maximum-likelihood phylogenetic tree was constructed using 34 *tams1*-like gene sequences determined in the present study. The *T*. *annulata* and *T*. *lestoquardi* sequences were retrieved from GenBank. P and N series numbers provided with the Sri Lankan sequences indicate animal IDs from Polonnaruwa and Nuwara Eliya, respectively. The Sri Lankan sequences clustered together and formed a sister clade to the common ancestor of the clades formed by the *T*. *annulata* and *T*. *lestoquardi* sequences from other countries.

Thirty-nine bovine blood DNA samples from the Polonnaruwa and Nuwara Eliya districts in Sri Lanka, which had tested positive on a *T*. *annulata*-specific PCR assay, were used in the present study^15^. From these samples, *tams1*-like gene sequences were successfully isolated from 34 samples (GenBank accession no. LC467536 - LC467569), including 28 from Polonnaruwa and 6 from Nuwara Eliya. The newly determined *tams1*-like nucleotide and translated amino acid sequences were highly conserved but shared low identity scores with *T*. *annulata* sequences (n = 125) from ten countries: Sudan, Tunisia, Mauritania, Bahrain, Turkey, Italy, Spain, Portugal, India, and China (Table 1). Multiple alignment of nucleotide sequences revealed that the TCC nucleotides, which were conserved across all *T. annulata* sequences at positions 598–600, were deleted in the Sri Lankan sequences (Fig. S1). The deletion of these nucleotides resulted in deletion of amino acid serine in Sri Lankan sequences (Fig. S2). Phylogenetically, the *tams1* nucleotide and amino acid sequences from GenBank formed multiple clades, and the clade formed by the *tams1* homologous sequences in *Theileria lestoquardi* was nested within the *T*. *annulata* clades (Figs 1 and S3). However, the Sri Lankan sequences formed a sister clade to the common ancestor of all *T. annulata* and *T*. *lestoquardi* sequences in GenBank (Figs 1 and S3).

**Table 1:** Nucleotide and amino acid identity scores (%) shared among the Sri Lankan sequences, among the *T. annulata* sequences, and between the *T*. *annulata* and Sri Lankan sequences.

Gene	Nucleotide sequences			Amino acid sequences		
	Sri Lankan	*T. annulata*	Between Sri Lankan and *T. annulata*	Sri Lankan	*T. annulata*	Between Sri Lankan and *T. annulata*
*tams1*	98.9–100	87.5–100	87.2–93.4	96.2–100	80.4–100	78.9–89.5
*tasp*	97.3–100	85.4–100	77.9–83.2	95.4–100	78.2–100	67.5–73.9
18S rRNA (short)	99.5–100	99.1–100	98.5–99.2	—	—	—
18S rRNA (long)	99.7–100	99.2–100	98.7–99.3	—	—	—
*cob*	99.4–100	98.5–100	87.2–88.0	98.8–100	97.1–100	82.5–84.8

**Figure 2:**
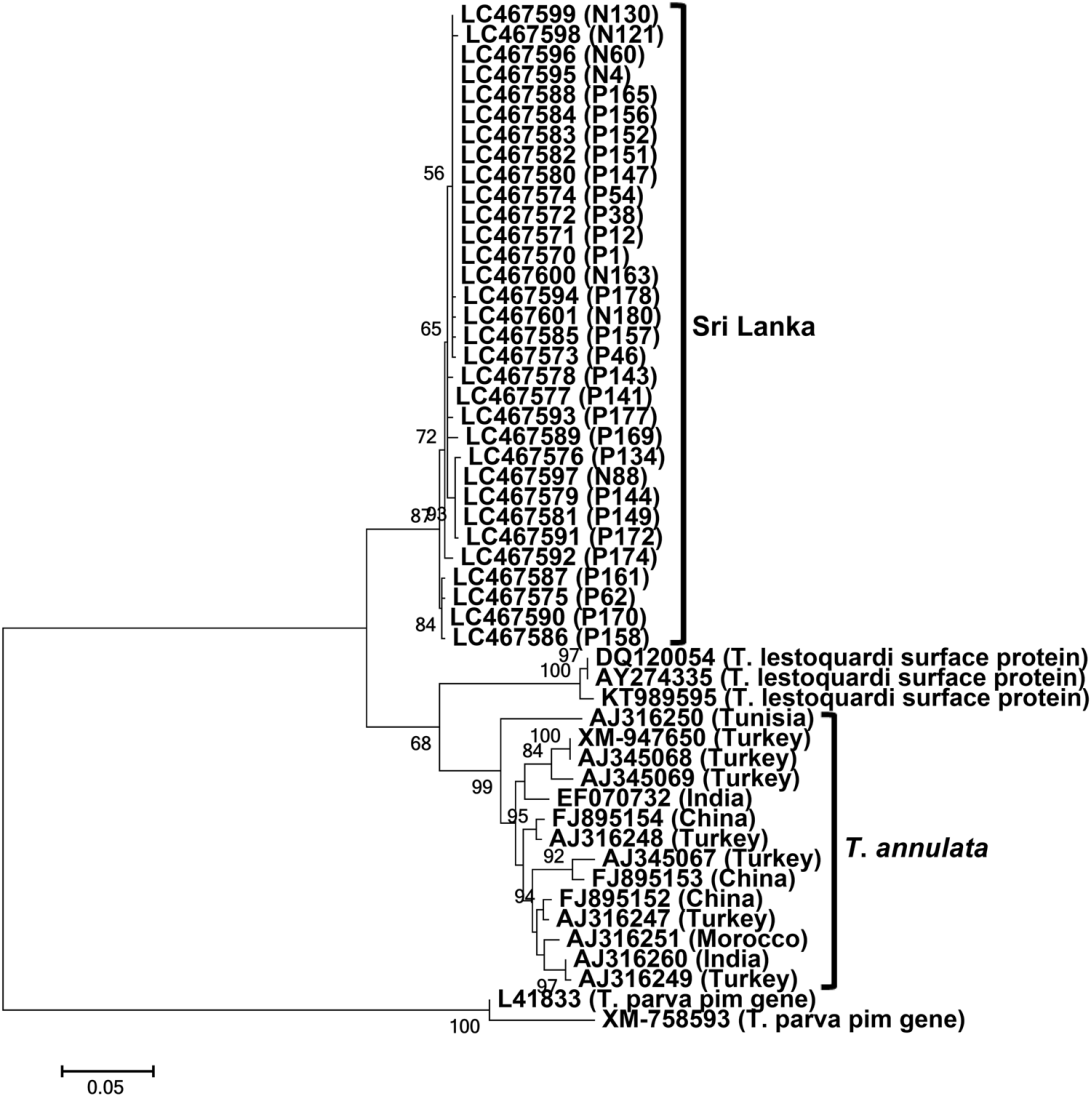
Phylogeny of the *tasp* gene. A maximum-likelihood phylogenetic tree was constructed using the coding sequences of 32 *tasp*-like gene sequences determined in the present study. The *T*. *annulata* and *T*. *lestoquardi* sequences were obtained from GenBank. P and N series numbers provided with the Sri Lankan sequences indicate animal IDs from Polonnaruwa and Nuwara Eliya, respectively. The Sri Lankan sequences clustered together and formed a sister clade to the common ancestor of the *T*. *lestoquardi* and *T*. *annulata* sequences from other countries.

The *tasp* gene sequences were isolated from 32 DNA samples (GenBank accession no. LC467570 - LC467601), including 25 and 7 sequences from Polonnaruwa and Nuwara Eliya, respectively. Length polymorphism was observed among the newly determined sequences, with 22 and 10 sequences containing 1,023 bp and 1,020 bp, respectively. As previously reported, the *tasp*-like sequences had two short introns^12^, which were removed from the primer-trimmed Sri Lankan sequences to obtain 839- and 842-bp coding sequences. The Sri Lankan coding nucleotide and amino acid sequences shared 97.3–100% and 95.4–100% identity scores, respectively, with each other and 77.9–83.2% and 67.5–73.9% identity scores, respectively, with the *T*. *annulata* sequences (n = 14) from Tunisia, Morocco, Turkey, India, and China (Table 1). The nucleotide and amino acid sequences at both ends of the *tasp* gene are usually conserved^12^. In contrast, the sequences in these regions, especially at the 5′ end in nucleotide sequences and N-terminal region in amino acid sequences, were poorly conserved between the Sri Lankan and *T*. *annulata* sequences (Figs S4 and S5). Phylogenetic analysis showed that that *T*. *lestoquardi* surface protein gene sequences formed a sister clade to the *tasp* sequences from GenBank, while all the Sri Lankan sequences formed a sister clade to the common ancestor of clades formed by the *T*. *annulata* and *T*. *lestoquardi* gene sequences (Fig. 2). A phylogeny constructed based on translated amino acid sequences had the same topology (Fig. S6). These findings suggested that the Sri Lankan isolates have genetic backgrounds that differed from those of *T*. *annulata*.

**Figure 3:**
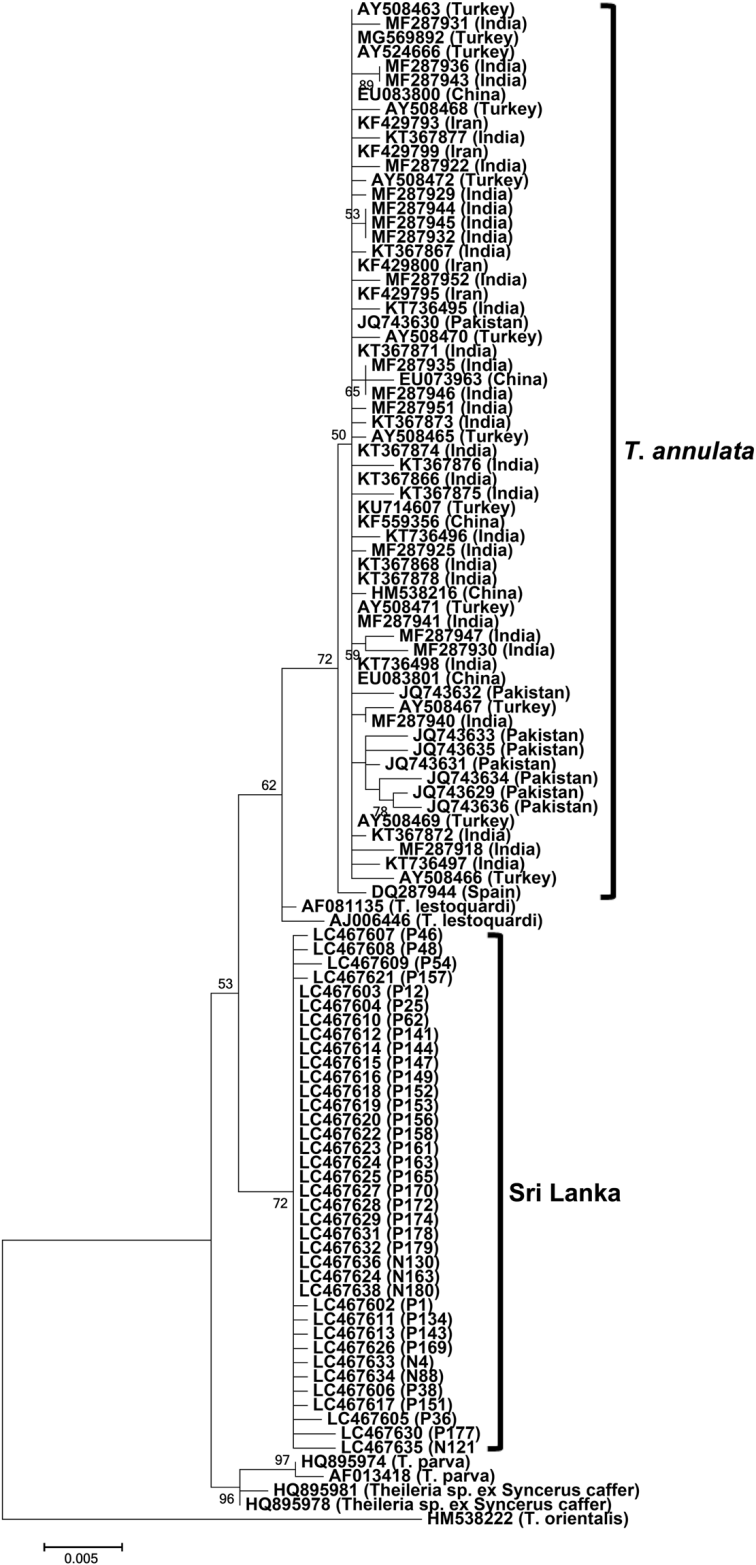
18S rRNA phylogeny. A maximum-likelihood phylogenetic tree was constructed using 37 short 18S rRNA gene sequences (1,130 bp) from Sri Lanka and the *T*. *annulata* and *T*. *lestoquardi* sequences from GenBank. P and N series numbers provided with the Sri Lankan sequences indicate animal IDs from Polonnaruwa and Nuwara Eliya, respectively. The Sri Lankan sequences clustered together and formed a sister clade to the common ancestor of the *T*. *lestoquardi* and *T*. *annulata* sequences from other countries.

**Figure 4:**
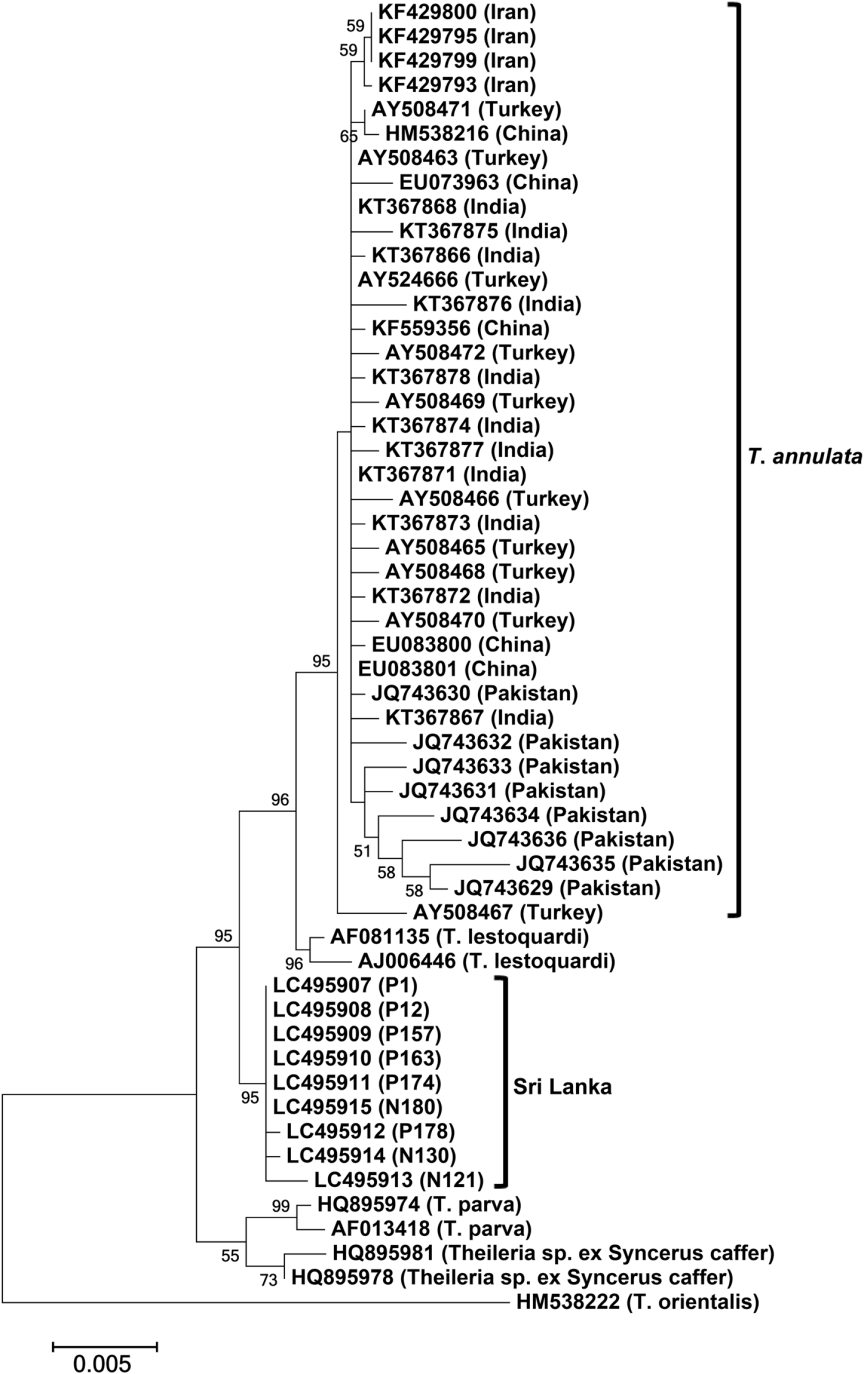
18S rRNA phylogeny. A maximum-likelihood phylogenetic tree was constructed using nine long 18S rRNA gene sequences (1,536 bp) from Sri Lanka and the *T*. *annulata* and *T*. *lestoquardi* sequences from GenBank. P and N series numbers provided with the Sri Lankan sequences indicate animal IDs from Polonnaruwa and Nuwara Eliya, respectively. The Sri Lankan sequences clustered together and formed a sister clade to the common ancestor of the *T*. *lestoquardi* and *T*. *annulata* sequences from other countries. Note that the bootstrap support for *T*. *annulata*, *T*. *lestoquardi*, and Sri Lankan clades and their separation improved compared with that in phylogeny constructed with short 18S rRNA sequences.

However, although the *tams1* and *tasp* genes are markers for analysing genetic diversity among *T*. *annulata*, these genes are unsuitable for evolutionary studies because they evolved under immune pressure. Therefore, we isolated 1,185-bp 18S rRNA fragment (1,130 bp after primer regions were trimmed) from 37 DNA samples (GenBank accession no. LC467602 - LC467638), including 31 from Polonnaruwa and 6 from Nuwara Eliya. These 18S rRNA sequences were highly conserved (99.5%–100% identities) and shared marginally low identities (98.0%–99.2%) with 63 *T*. *annulata* sequences from six countries, including Iran, Turkey, Spain, India, China, and Pakistan (Table 1). Phylogenetic analysis showed that the Sri Lankan sequences formed a monophyletic sister clade to the common ancestors of the *T*. *annulata* and *T*. *lestoquardi* 18S rRNA sequences (Fig. 3). However, the clades were supported with low bootstrap values. Thus, we isolated relatively long fragment (1,536 bp after primer regions were trimmed) of 18S rRNA sequences (GenBank accession no. LC495907 - LC495915), using a set of universal forward and reverse primers, from nine samples with single infection. In these sequences, we identified ten unique single-nucleotide polymorphisms (SNPs), including a deletion and nine substitutions compared with the GenBank-derived *T*. *annulata* sequences (Fig. S7). The topologies of phylogenetic trees constructed with short and long 18S rRNA sequences were similar, but the bootstrap support for clades drastically increased in the later (Fig. 4).

**Figure 5:**
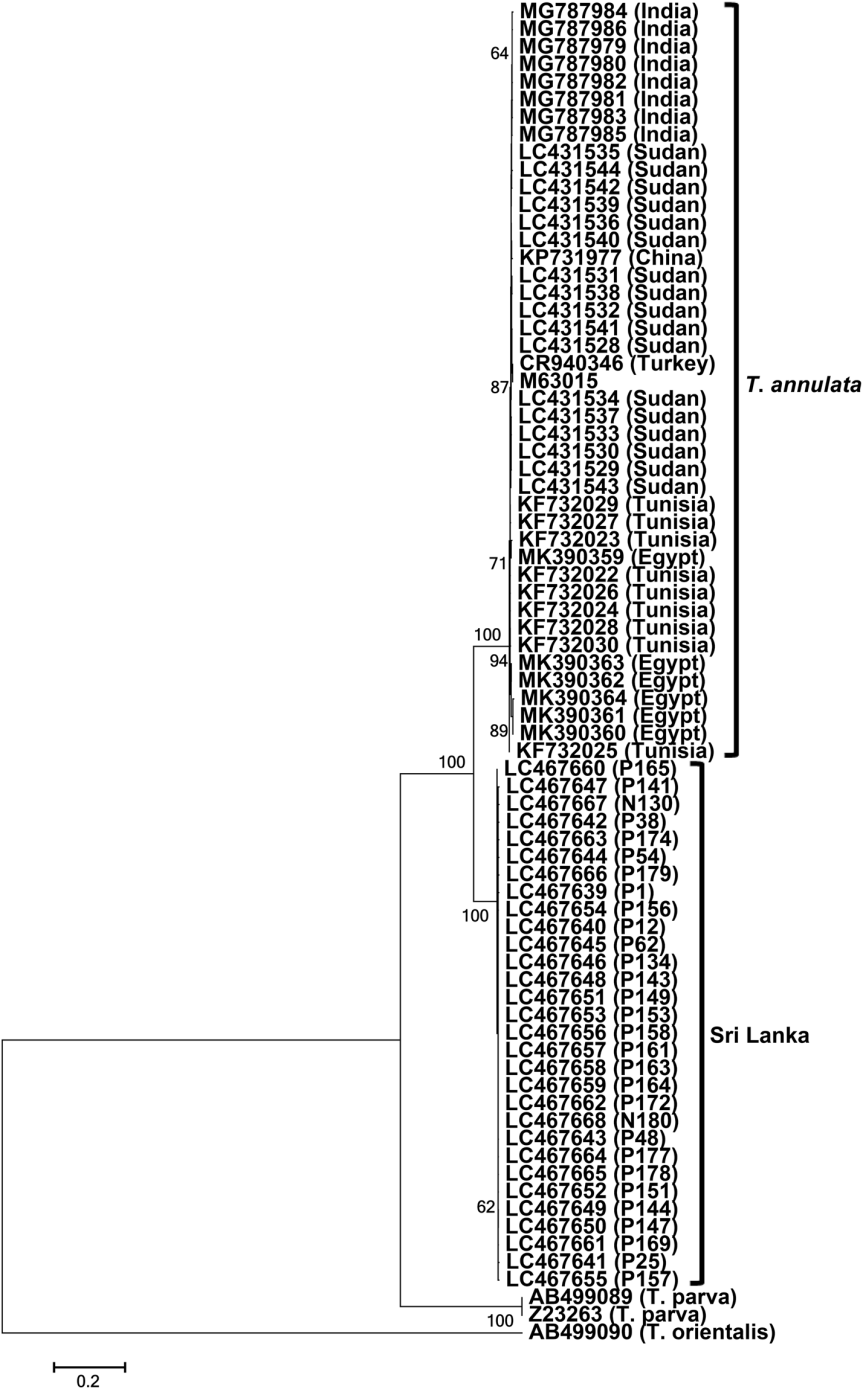
Phylogeny of the *cob* gene. A maximum-likelihood phylogenetic tree was constructed using 30 *cob* gene sequences from Sri Lanka and the *T*. *annulata* sequences from GenBank. P and N series numbers provided with the Sri Lankan sequences indicate animal IDs from Polonnaruwa and Nuwara Eliya, respectively. The Sri Lankan sequences clustered together and formed a sister clade to the *T*. *annulata* sequences from other countries.

Although the phylogenetic position and unique SNPs may identify the Sri Lankan isolates as a new *Theileria* sp., this assumption remains inconclusive as the 18S rRNA sequences shared high identity scores with the *T*. *annulata* sequences. Therefore, we obtained 30 *cob* gene sequences (GenBank accession no. LC467639 - LC467668), including 28 from Polonnaruwa and 2 from Nuwara Eliya, and compared them with the 43 *T*. *annulata* sequences from Sudan, Tunisia, Egypt, Turkey, India, and China. The *T*. *annulata cob* nucleotide and translated amino acid sequences from these countries were highly conserved, sharing 98.5–100% and 97.1–100% identity scores, respectively (Table 1). In contrast, these sequences shared only 87.2–88.0% and 82.5–84.8% identities with the Sri Lankan *cob* nucleotide and amino acid sequences, respectively (Figs S8 and S9). In addition, the Sri Lankan *cob* gene sequences were characterized by 122 unique SNPs compared with the GenBank-derived *T*. *annulata* sequences (Fig. S8). Phylogenetic analysis showed that the Sri Lankan *cob* nucleotide as well as amino acid sequences clustered together and formed a sister clade to the *T*. *annulata* clade (Figs 5 and S10), further supporting our assumption that the *Theileria* sp. from the cattle in Sri Lanka is not *T*. *annulata* but a *Theileria* sp. closely related to *T*. *annulata*.

## Discussion

The unique polymorphisms, low identity scores shared with the *T*. *annulata* sequences, and distinct phylogenetic positions collectively indicated that the parasite species from Sri Lanka, identified via PCR as *T*. *annulata*, was indeed a new *Theileria* sp. closely related to *T*. *annulata*.

Although the present study was initiated to analyse the genetic diversity of *T*. *annulata* in Sri Lanka, the *tams1*- and *tasp*-like sequences from the Sri Lankan samples diverged from known *T*. *annulata* sequences. However, there is no bootstrap support for the *T*. *annulata* clade in *tams1* phylogeny. The genetic diversity of *tams1* is thought to be due to random intragenic recombination, which results in a mosaic pattern of diversity^16^. This could explain the lack of bootstrap support for the *T*. *annulata* clade. In addition, there are no bootstrap values for the separation of Sri Lankan clades in both *tams1* and *tasp* phylogenies due to lack of additional outgroup sequences. In *tams1* phylogeny, addition of further outgroup sequences, such as those from *Theileria taurotragi* and *Theileria* *orientalis*, however resulted in altered topology due to deletion of regions shared between *T*. *annulata* and *T*. *lestoquardi* from the alignment during phylogeny construction. Therefore, we refrained from using additional outgroup sequences in the *tams1* phylogeny. For the *tasp* phylogeny, we could not find suitable additional outgroup sequences.

Next, short 18S rRNA sequences from the Sri Lankan samples were analysed. In phylogeny, the Sri Lankan sequences occurred in a clade unrelated to *T*. *annulata*. However, *T*. *annulata*, *T*. *lestoquardi*, and Sri Lanka clades and their separation were not supported by bootstrap values. Phylogenies constructed with short 18S rRNA sequences are sometimes characterized by low bootstrap values, which usually improve when longer sequences are used^17^. Therefore, we isolated relatively longer 18S rRNA sequences and used them for phylogeny construction. The topology of this phylogeny was similar to that constructed with short 18S rRNA sequences, but the bootstrap support for each clade and their separation drastically improved. *Theileria lestoquardi* is thought to have recently evolved from *T*. *annulata*, following a host jump from cattle/buffalo to sheep/goat^18^. Despite the similarities between *T*. *lestoquardi* and *T*. *annulata*, they are considered as distinct parasite species because of the stringent host-specificity of the former as confirmed by *in vitro* and *in vivo* experiments^19^. Therefore, our observation that the Sri Lankan clade in 18S rRNA phylogeny formed sister clade to the common ancestor of *T*. *lestoquardi* and *T*. *annulata* indicated that the Sri Lankan samples were infected with a new *Theileria* sp.

However, in terms of identity scores, the Sri Lankan 18S rRNA sequences differed only marginally from the *T*. *annulata* sequences. High identities are common between 18S rRNA sequences from different *Theileria* species possibly due to recent speciation events^20^. The hypervariable region of the 18S rRNA from *T. lestoquardi* differs by only two nucleotides compared with *T*. *annulata*^21^. Similarly, 18S rRNA is highly conserved between *Theileria* *parva* and *Theileria* sp. (buffalo)^22^. However, mitochondrial gene sequences adequately discriminated these parasite species^20^. Among the *T*. *annulata* mitochondrial genes, only the *cob* sequences were available in sufficient numbers in the GenBank. Therefore, we obtained the *cob* sequences from the Sri Lankan samples and compared them with those from *T*. *annulata* isolates in different countries^23,24^. The Sri Lankan sequences were conserved but highly diverse compared with the similarly conserved *T*. *annulata* sequences. To the best of our knowledge, such a high diversity of mitochondrial genes was never reported for any species of protozoan parasites. These observations suggest that the parasite species, which had been previously identified as *T*. *annulata* in Sri Lanka, is a novel *Theileria* sp.

The *tams1*-like gene sequences in the new *Theileria* sp. were highly conserved. Similarly, although the *tasp*-like sequences from Sri Lanka had some diversity, the gene was relatively conserved compared with the *T. annulata* sequences. Low genetic diversity among *Theileria* parasites is thought to be due to their short evolutionary histories. For example, the genetic diversity of *T*. *parva* in buffalo is greater than that in cattle because this pathogen has evolved longer in the former host than in the later^8^. Similarly, in *T*. *lestoquardi*, the genetic diversity of the *tams1* homologous gene is lower than that of the *tams1* gene because *T*. *lestoquardi* is thought to have recently evolved^18^. Therefore, there is a possibility that the new *Theileria* sp. was introduced in Sri Lanka relatively recently as a clonal population. If so, countries other than Sri Lanka may also be endemic to this *Theileria* sp. Epidemiological surveys to detect the new *Theileria* sp. in cattle from other countries may clarify this.

Previously, phylogenetic analyses conducted using 18S rRNA sequences found that the transforming *Theileria* species, including *T*. *parva*, *T*. *annulata*, *T*. *taurotragi*, *Theileria* sp. (buffalo), and *T*. *lestoquardi*, have a common ancestor^20,25^. Therefore, detecting the new *Theileria* sp. as a sister clade to the common ancestor of the *T*. *annulata* and *T*. *lestoquardi* sequences infers that the *Theileria* sp. may also be a transforming *Theileria*. However, isolation and *in vitro* cultivation of the schizont stage, together with genome sequencing analysis to identity the host cell transformation-related genes^26^, are essential to confirm whether the new *Theileria* sp. is a transforming species.

In conclusion, the present study, which aimed to analyse the genetic diversity of *T*. *annulata* in Sri Lanka, unexpectedly revealed a novel *Theileria* sp. However, the morphological, clinical, and pathological distinctions of this novel *Theileria* sp. are yet to be investigated. Therefore, the new species was provisionally designated as *Theileria* sp. Yokoyama.

## Methods

### DNA samples

Thirty-nine archived DNA samples from blood collected from cattle in Polonnaruwa (n = 32) and Nuwara Eliya (n = 7) in June 2014 were used in the present study^15^. All animals were apparently healthy during sampling. DNA samples were prepared from 200 µl of whole blood using a commercial kit (Qiagen, Hilden, Germany) following the manufacturer’s instructions. All DNA samples were positive for *T*. *annulata* via PCR assay based on the *tams1* gene^15,27^.

### PCR amplification, cloning, and sequencing of *tams1*, *tasp*, 18S rRNA, and *cob*

The 18S rRNA and the *tams1*, *tasp*, and *cob* gene sequences were amplified via PCR assays using primer sets designed in the present study. Briefly, three sets of forward and reverse primers were designed to amplify the full-length *tams1*, *tasp*, and *cob* genes using multiple alignments based on the *T*. *annulata* gene sequences retrieved from GenBank (Table 2). For the 18S rRNA, *T*. *annulata*-specific forward and reverse primers were designed to amplify an 1,185-bp fragment based on multiple alignment of the 18S rRNA sequences from bovine *Babesia* and *Theileria* species, including *B*. *bovis*, *B*. *bigemina*, and *T*. *orientalis*, because some of the *T*. *annulata*-positive DNA samples were coinfected with these species^15^. In addition, previously described universal forward^28^ and reverse^29^ primers were used to amplify a long fragment of 18S rRNA (≈1600 bp) from nine DNA samples with single infection. Each PCR assay was conducted in a 10-µl reaction mixture containing 1 µl of *T*. *annulata*-positive genomic DNA, 1 × PCR buffer (10 × PCR buffer, Applied Biosystems, Branchburg, NJ, USA), 200 µM of each dNTP (Applied Biosystems), 0.5 µM of each forward and reverse primer, 0.1 µl of 5 U/µl taq polymerase (Applied Biosystems), and 5.9 µl distilled water. The reaction mixture was then subjected to an initial predenaturation step at 95 °C for 5 min, followed by 45 cycles each of denaturation at 95 °C for 30 sec, an annealing step at the appropriate temperature (listed in Table 1) for 30 sec, and an extension step at 72 °C for 2 min. After a final elongation step at 72 °C for 7 min, the PCR products were resolved by 1.5% agarose gel electrophoresis, stained with ethidium bromide, and visualized under ultraviolet (UV) illumination. PCR bands with appropriate sizes were gel-extracted, ligated to PCR 2.1 plasmid vectors, and sequenced using an ABI PRISM 3100 genetic analyser (Applied Biosystems). All sequences generated in the present study were trimmed to remove the primer regions before conducting the sequencing and phylogenetic analyses.

**Table 2:** List of primers, annealing temperatures, and sequence sizes.

Gene	Primer sequence (5′-3′)		Annealing T (°C)	Amplicon size (bp)	Size (bp) after primers were removed
	Forward	Reverse			
*tams1*	ATGTTGTCCAGGACCACCCTC	TTAAAGGAAGTAAAGGACTGATGAGAAG	56	843	794
*tasp*	ATGAAATTCTTCTACCTTTTTGTTCTATTTCC	TTAACAACAATCTTCGTTAATGCGAG	65	1020 and 1023	962 and 965
18S rRNA (short)	AGGGCTAATACATGTTCGAGGCCATTT	TCCCTCTAAGAAGCGATAACGGGACAGT	64	1185	1130
18S rRNA (long)	AAGCCATGCATGTCTAAGTATAAGCTTTT	GAATAATTCACCGGATCACTCG	56	1587	1536
*cob*	ATGAATTTGTTTAACTCACATTTGC	TTATGCACGAACTCTTGCAGAG	55	1092	1045

Primers designed in the present study were used to amplify *tams1*, *tasp*, short fragment of 18S rRNA, and *cob* genes, while long fragment of 18S rRNA was amplified using previously described forward^28^ and reverse^29^ primers.

### Gene sequences from GenBank

The *T*. *annulata tams1* gene sequences (n = 125) and coding sequences (n = 14) of the *tasp* gene with 100% coverage to the Sri Lankan sequences were retrieved from GenBank. Similarly, 63 and 38 *T*. *annulata* 18S rRNA sequences with 100% coverage to the Sri Lankan short and long 18S rRNA sequences, respectively, were also retrieved from GenBank. Nucleotide sequences with degenerate bases or an “n” symbol were omitted. In addition, some 18S rRNA sequences registered as derived from *T*. *annulata* (MF287917, MF287919, MF287920, MF287924, MF287934, MF287937, MF287939, MF287942, MF287948, MF287949, MF287950, KT736499, KT367869, and KT367870) were ignored because they shared high identity scores with *T*. *orientalis* compared with *T*. *annulata* in our BLAST search. The GenBank-derived *T*. *annulata* nucleotide and amino acid sequences were aligned with the Sri Lankan sequences, and then trimmed to remove the excess nucleotides or amino acids. For the *cob* gene, the *T*. *annulata* sequences (n = 43) from GenBank with 98% coverage to the Sri Lankan sequences were obtained. These *cob* nucleotide sequences and translated amino acid sequences were aligned with the Sri Lankan sequences. Excess nucleotides or amino acids in the alignment were removed at the 3ʹ end or C-terminal based on the Sri Lankan sequences and at the 5ʹ end or N-terminal based on the Sudanese sequences.

### Calculation of identity scores

Alignments of the 18S rRNA sequences and nucleotide and amino acid sequences of *tams1*, *tasp* (coding region), and *cob* genes, which contained both the GenBank-derived *T*. *annulata* and Sri Lankan sequences, were analysed using MatGAT software^30^ to calculate the identity scores among the Sri Lankan sequences, among the GenBank-derived sequences, and between the Sri Lankan and GenBank-derived sequences.

### Phylogenetic analyses

18S rRNA sequences and nucleotide and amino acid sequences of *tams1*, *tasp* (coding region), and *cob* genes from GenBank and those determined in the present study were subjected to multiple alignment using Multiple Alignment using Fast Fourier Transform (MAFFT) online software^31^. The aligned sequences were analysed using Molecular Evolutionary Genetics Analysis (MEGA), version 6.0 software^32^ to predict the best-fitting substitution models based on the lowest Akaike information criterion (AIC) values. Subsequently, using nucleotide sequences, five maximum likelihood phylogenetic trees were constructed based on general time reversible^33^ (*tams1*, *tasp*, and 18S rRNA-short) and Tamura-Nei^34^ (18S rRNA-long and *cob*) substitution models, using MEGA software^32^. Similarly, three maximum likelihood phylogenetic trees were constructed using amino acid sequences based on Dayhoff^35^ (TAMS1), Whelan And Goldman^36^ (TASP), and JTT^37^ (COB) substitution models. All positions containing gaps were removed from the alignments before phylogeny construction.

### Ethical approval

The Animal Care and Use Committee of Obihiro University of Agriculture and Veterinary Medicine, Japan, approved all animal procedures (approval number: 29–53). All experiments were carried out in accordance with the Fundamental Guidelines for Proper Conduct of Animal Experiment and Related Activities in Academic Research Institutions coming under the Ministry of Education, Culture, Sports, Science and Technology, Japan.

## Supplementary information

Supplementary information

## Data Availability

The materials and datasets generated in the present study are available from the corresponding author upon reasonable request.
