# Supplementary material for: Discovery of a new Theileria sp. closely related to Theileria annulata in cattle from Sri Lanka
Source: Sci Rep. 2019 Nov 6;9:16132. doi: 10.1038/s41598-019-52512-y (PMC6834563; doi:10.1038/s41598-019-52512-y)
Supplement: Supplementary file 1 — Supplementary information [file 41598_2019_52512_MOESM1_ESM.pdf]

Discovery of a new *Theileria* sp. closely related to *Theileria annulata* in cattle from Sri Lanka

Supplementary Information

Thillaiampalam Sivakumar[1], Shiori Fujita[1], Bumduuren Tuvshintulga[1], Hemal Kothalawala[2], Seekkuge Susil Priyantha Silva[2], Naoaki Yokoyama[1,3*]

[1]National Research Center for Protozoan Diseases, Obihiro University of Agriculture and Veterinary Medicine, Hokkaido, Japan
[2]Veterinary Research Institute, Peradeniya, Sri Lanka
[3]OIE reference laboratory for bovine babesiosis and equine piroplasmosis, National Research Center for Protozoan Diseases, Obihiro University of Agriculture and Veterinary Medicine, Hokkaido, Japan

[*]**Corresponding author:** N. Yokoyama, National Research Center for Protozoan Diseases, Obihiro University of Agriculture and Veterinary Medicine, Inada-cho, Obihiro, Hokkaido 080-8555, Japan. Tel: +81 155 49 5649; Fax: +81 155 49 56

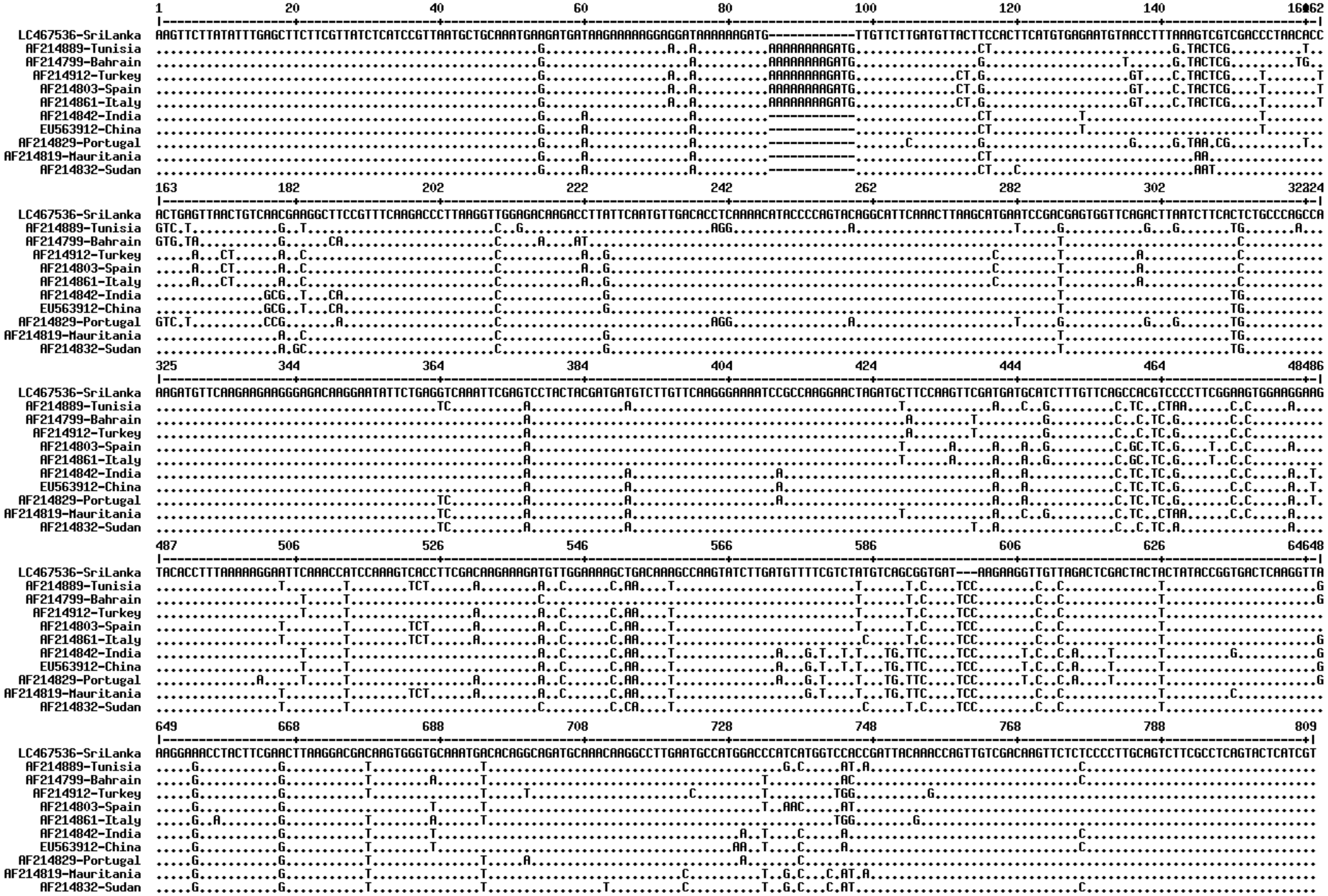

Fig. S1. Multiple alignment of *tams1* sequences. A representative *tams1*-like sequence determined in this study was aligned with *tams1* sequences from different countries. Nucleotide bases identical to the reference sequence are indicated by dots, while dashes denote nucleotide deletions. The TCC nucleotides, which were conserved among all *T. annulata* sequences at positions 598–600, were deleted in the Sri Lankan sequences.

```
                    1                 20                  40                  60                  80                 100                 120            135
                    |------------------+-------------------+-------------------+-------------------+-------------------+-------------------+--------------|
  LC467536-SriLanka KFLYLSFFVISSVNAANEDDKKKEDKKD----VVLDVTSTSCENVTFKVVDPNTTELTVNEGFRFKTLKVGDKTLFNVDTSKHTPVQAFKLKHESDEWFRLNLHSAQPKMFKKKGDKEYSEVKFESYYDDVLFKG
   AF214889-Tunisia ........................E...EKKD......L........DTR...SVV...KD...................G.....K......D.E.....D..A................S...T...E.....
   AF214799-Bahrain ........................E...EKKD......A........DTR...AVV...K..H........E.N..............................P.....................T.........
    AF214912-Turkey ........................E...EKKD.....LA......V.HTR.S.....L.KD......................................K....P.....................T.........
     AF214803-Spain ........................E...EKKD.....LA......V.HTR.S.....L.KD......................................K....P.....................T.........
     AF214861-Italy ........................E...EKKD.....LA......V.HTR.S.....L.KD......................................K....P.....................T.........
     AF214842-India ...................E....E...----......L...D........S.......AD.H.........................................A.....................T...E.....
     EU563912-China ...................E....E...----......L...D........S.......AD.H.........................................A.....................T...E.....
  AF214829-Portugal ...................E....E...----......A......A.DNR...SVV...P..Y.................G.....K......D.E.....D..A................S...T...E.....
AF214819-Mauritania ...................E....E...----......L.........N..........KD...........................................A................S...T...E.....
     AF214832-Sudan ...................E....E...----......L.........N..........KG...........................................A................S...T...E.....

                    136            155                 175                 195                 215                 235                 255            270
                    |------------------+-------------------+-------------------+-------------------+-------------------+-------------------+--------------|
  LC467536-SriLanka KSAKELDASKFDDASLFSHVPFGSGRKYTFKKEFKPSKVTFDKKDVGKADKAKYLDVFVYVSGD-KKVVRLDYYYTGDSRLKETYFELKDDKWVQMTQADANKALNAMDPSWSTDYKPVVDKFSPLAVFASVLIV
   AF214889-Tunisia .......V...E.PA..TSAN..T.K......D......L.E..E...PNN........F..A.S........F......................S.............A..S.....................
   AF214799-Bahrain ..............A..TPSA..T..............................F..A.S........F......................S............S...P.....................
    AF214912-Turkey ..............A..TPSA..T.................E..E...PNN........F..A.S........F......................S.S..........S..LA.....................
     AF214803-Spain .......V...E.TA..TASA..T.K......D......L.E..E...PNN........F..A.S........F......................S............SN..S.....................
     AF214861-Italy .......V...E.TA..TASA..T........D......L.E..E...PNN...........A.S........F......................S...............LA..E.....................
     AF214842-India ...........E.T...TSSA..T.KM...................E...PNN....E.V.F.GS.S..F.K.Y.F.............................NS.........................
     EU563912-China ...........E.T...TSSA..T.KM...................E...PNN....E.V.F.GS.S..F.K.Y.F............................INS.........................
  AF214829-Portugal ...........E.T...TSSA..T.KM...............E..E...PNN....E.V.F.GS.S..F.K.Y.F......................S.T.........N..........................
AF214819-Mauritania .......V...E.PA..TSAN..T.K......D......L.E..E...PNN......V.F.GS.S........F......................S........T...SA.PS.....................
     AF214832-Sudan ...........E.....TPST....K......D...............PNN...........A.S........F......................S........T...SA.PS.....................
```

Fig. S2. Multiple alignment of TAMS1 amino acid sequences. A representative TAMS1-like sequence determined in this study was aligned with TAMS1 sequences from different countries. Amino acid residues identical to the reference sequence are indicated by dots, while dashes denote amino acid deletions. A serine amino acid residue, which was conserved among all *T. annulata* sequences at position 200, was deleted in the Sri Lankan sequences.

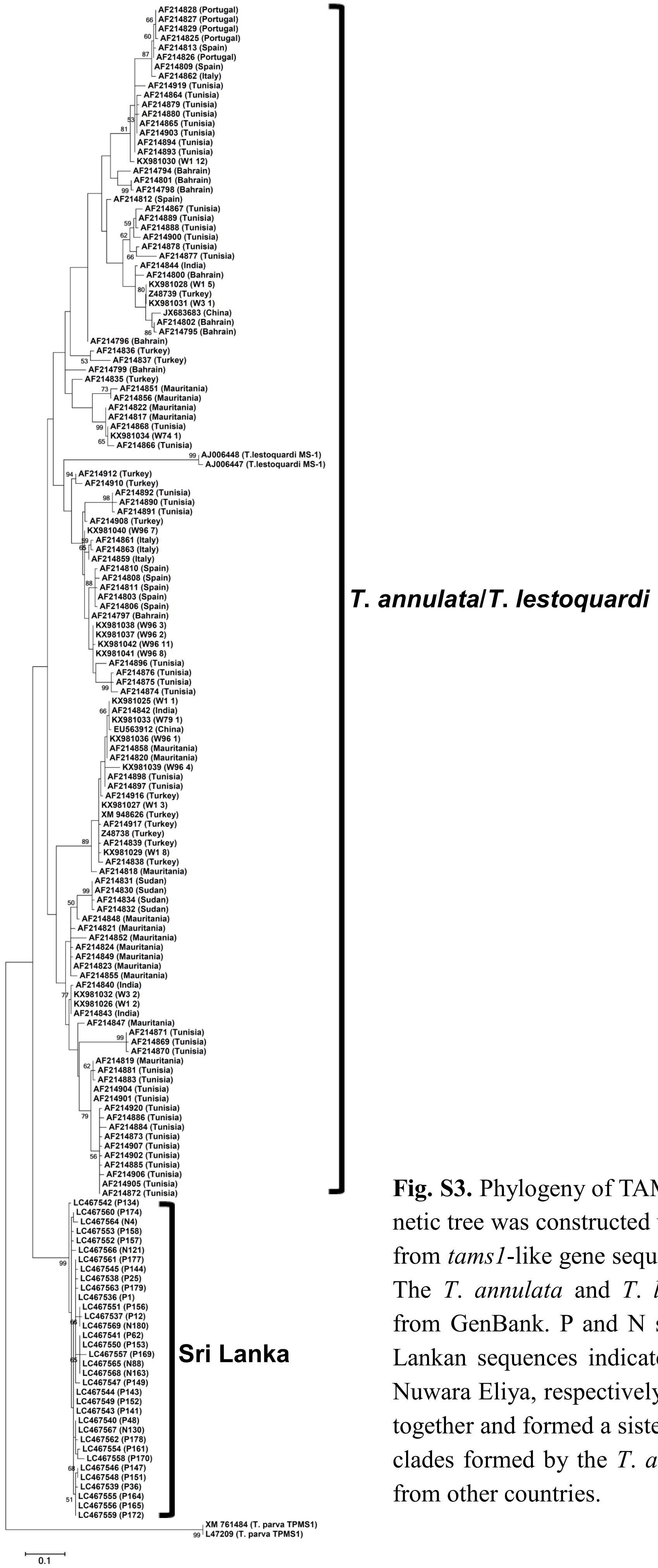

Fig. S3. Phylogeny of TAMS1. A maximum-likelihood phylogenetic tree was constructed using amino acid sequences translated from *tams1*-like gene sequences determined in the present study. The *T. annulata* and *T. lestoquardi* sequences were retrieved from GenBank. P and N series numbers provided with the Sri Lankan sequences indicate animal IDs from Polonnaruwa and Nuwara Eliya, respectively. The Sri Lankan sequences clustered together and formed a sister clade to the common ancestor of the clades formed by the *T. annulata* and *T. lestoquardi* sequences from other countries.

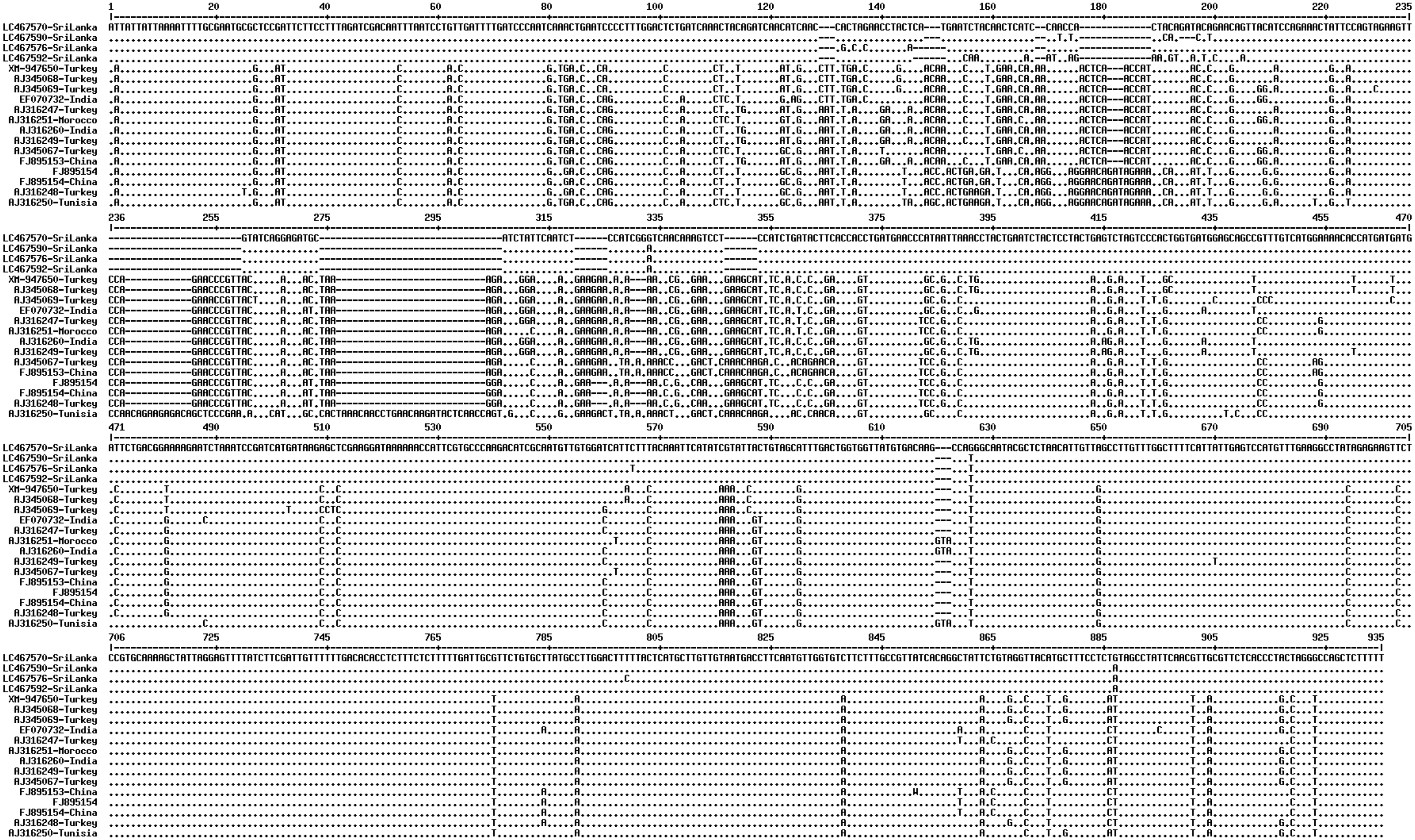

Fig. S4. Multiple alignment of *tasp* sequences. Four representative *tasp*-like sequences determined in this study were aligned with *tasp* sequences from different countries. Nucleotide bases identical to the reference sequence are indicated by dots, while dashes denote nucleotide deletions. Nucleotides at both ends of the *tasp* sequences are conserved but are diverged from the Sri Lankan sequences.

```
                  1                 20                  40                  60                  80                 100                 120                 140             156
                  |------------------+-------------------+-------------------+-------------------+-------------------+-------------------+-------------------+---------------|
LC467570-SriLanka LLLKFCECAPILPLDRQFNPVDFDPNQTESPLDSDQTTDQHQPLEPTHEST-------THPTTTDTEQLHPETIPVEVVSGDASIQSPSG-----------------------QQSPPSDTSPPDEPIIKPTESTPTESSPTGDGAAVCHGKHHDD
LC467576-SriLanka ...........................................PQ..-...-------...............................D-----------------------...........................................
LC467590-SriLanka ...................................................-------...S.-QP.......................D-----------------------...........................................
LC467592-SriLanka ...............................................EQT.-------Q.-.ESEIQ......................D-----------------------...........................................
 XM_947650-Turkey I.......G.F......L..I.....DDQH...P..LI..IE.S.QPAQQEPIEPQQP.Q.S.-EP.E.Q...VT...P-EPVTSEE.KE------------SDQTEEQKH-EEPEASPAPE.V...AVHA......KA.SS.............Y
  AJ345068-Turkey I.......G.F......L..I.....DDQH...P..LI..IE.S.QPAQQEPIEPQQP.Q.S.-EP.E.Q...VT...P-EPVTSEE.KE------------SDQTEEQKH-EEPEASPAPE.V...AVHA......KA.SS.............Y
  AJ345069-Turkey I.......G.F......L..I.....DDQH...P..LI..IE.S.QPAQQEPIEPQQP.Q.S.-EP.E.E...VT...P-ETVTSEE.KE------------SDQTEEQKH-EEPEASPAPE.V...AVQ.......KA.SS..A..P.......H
   EF070732-India I.......G.F......L..I.....DDQQ...PN.LI..EE.S.QPTQQEPIEPQQP.Q.S.-EP.E.D...VT...P-EPVTSEESKE------------SDQTEEQKH-EEPEASPVPE.V...AVQA......KA.SS.E.............
  AJ316247-Turkey I.......G.F......L..I.....DDQQ...PN.LM..IEQSQEDTQQEPIEPQQP.Q.S.-EP.E.Q...VT...P-EPVTSEE.KE------------SDQTEEQKH-EEPEASPVPE.V...PVQ.......KA.SS.....P........
   FJ895152-China I.......G.F......L..I.....DDQQ...PN.LM..IEQSQEDTQQEPIEPQQP.Q.S.-EP.E.Q...VT...P-EPVTSEE.KE------------SDQTEEQKH-EEPEASPVPE.V...PVQ.......KA.SS.....P........
 AJ316251-Morocco I.......G.F......L..I.....DDQQ...PN.LI..VEQSQEDTQQEPIEPQQP.Q.S.-EP.E.E...VT...P-EPVTSEE.KE------------STQTEEQKH-EEPEASPVPE.V...PVQ.......KA.SS.....P........
   AJ316260-India I.......G.F......L..I.....DDQQ...PN.LM..IEQSQEDTQQEPIEPQQP.Q.S.-EP.E.Q...VT...P-EPVTSEE.KE------------SDQTEEQKH-EEPEASPAPE.V...AVHA......KA.SS.E.............
  AJ316249-Turkey I.......G.F......L..I.....DDQQ...PN.LM..IEQSQEDTQQEPIEPQQP.Q.S.-EP.E.Q...VT...P-EPVTSEE.KE------------SDQTEEQKH-EEPEASPAPE.V...AVHA......KA.SS.E.............
  AJ345067-Turkey I.......G.F......L..I.....DDQQ...PN.LI..AEQSQESTQQESIEPQQP.Q.S.-EP.E.E...VT...P-EPVTSEE.KE------------STQTEEPTETQDSKQEPTEQ.V...PVQ.......KA.SS.....P..E.....
   FJ895153-China I.......G.F......L..I.....DDQQ...PN.LM..IEQSQEDTQQEPIEPQQP.Q.S.-EP.E.E...VT...P-EPVTSEE.KE------------STQTEEPTETQDSKQEPTEQ.V...PVQ.......KA.SS.....P..E.....
   FJ895154-China I.......G.F......L..I.....EDQQ...PN.LI..AEQSQEPIQP.DESPQEQEQIE.QES.E.E...VT...P-EPVTSEESKE------------STQTEQKP--E.PEASPAPE.V...PVQ.......KA.SS.....P........
  AJ316248-Turkey I.......G.F......L..I.....EDQQ...PN.LI..AEQSQEPIQP.EESPQEQEQIE.QES.E.E...VT...P-EPVTSEESKE------------STQTEQKP--E.PEASPAPE.V...PVQ.......KA.SS.....P........
 AJ316250-Tunisia I.......G.F......L..I.....DDQQ...PN.LI..AEQSQEPIQA.EESPQEQEQIE.QES.E.E..SVS...PTEETAPEESIEPTKQPEQDTQPVATQAEDSTETQDSKQE.TQQ.V...A.Q.......KA.SS...S.P........

                  157              176                 196                 216                 236                 256                 276                 296            311
                  |------------------+-------------------+-------------------+-------------------+-------------------+-------------------+-------------------+--------------|
LC467570-SriLanka DSDGKESKSDHDKSSKDKKPFVPKTSQCCGSFFTNSYRITVAFDWWLCDK-PGQYALTLLALFGFSLLSPCLKAYREVLRAKAIRSFIFDCFLTHLFLFLIAFCAYALDFLLMLVVMTFNVGVFFAVITGYSVGYMLSSVAYSTLRSHPTRASSF
LC467576-SriLanka ..................................................-.W......................................................................................I...............
LC467590-SriLanka ..................................................-.W......................................................................................I...............
LC467592-SriLanka ..................................................-.W......................................................................................I...............
 XM_947650-Turkey .............RP................Y.....K............-.W..............................................................................T...LV..L.........A.S...
  AJ345068-Turkey .............RP................Y.....K............-.W..............................................................................T...LV..L.........A.S...
  AJ345069-Turkey ...........Y.PP...............A......K............-.W..............................................................................T...LV..L.........A.S...
   EF070732-India ......P......RP...............P......K.V..........-.W..............................................................................T...L...L.........A.S...
  AJ316247-Turkey .............RP...............P......K.V..........-.W..............................................................................T...L...L...........S...
   FJ895152-China ......P......RP...............P......K.V..........-.W..............................................................................T...L...L...........S...
 AJ316251-Morocco .............RP......................K.V..........V.W..............................................................................T...LV..L.........A.S...
   AJ316260-India .............RP...............P......K.V..........V.W..............................................................................T...LV..L.........A.S...
  AJ316249-Turkey .............RP...............P......K.V..........-.W.............F................................................................T...LV..L.........A.S...
  AJ345067-Turkey .............RP......................K.V..........-.W..............................................................................T...LV..L.........A.S...
   FJ895153-China .............RP...............P......K.V..........-.W..........................................................................X...T...L...L...........S...
   FJ895154-China .............RP...............P......K.V..........-.W..............................................................................T...L...L...........S...
  AJ316248-Turkey .............RP...............P......K.V..........-.W..............................................................................T...L...L.........A.S...
 AJ316250-Tunisia ......P......RP...............P......K.V..........V.W..............................................................................T...LV..L.........A.S...
```

Fig. S5. Multiple alignment of TASP amino acid sequences. Four representative TASP-like sequences determined in this study were aligned with TASP sequences from different countries. Amino acid residues identical to the reference sequence are indicated by dots, while dashes denote amino acid deletions. Amino acid residues at both ends of the TASP sequences are conserved but are diverged from the Sri Lankan sequences.

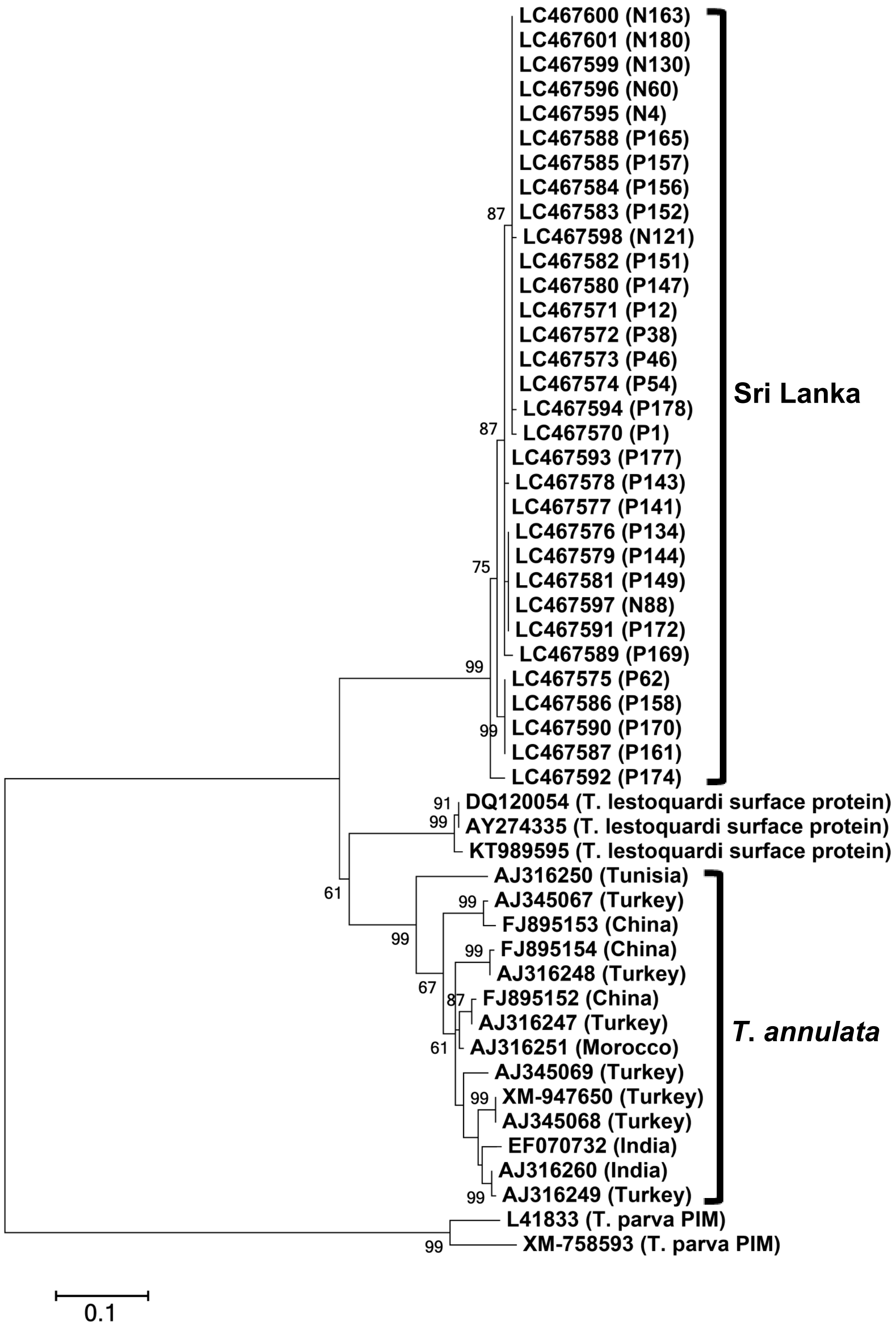

Fig. S6. Phylogeny of the TASP. A maximum-likelihood phylogenetic tree was constructed using amino acid sequences of 32 *tasp*-like gene sequences determined in the present study. The *T. annulata* and *T. lestoquardi* sequences were obtained from GenBank. P and N series numbers provided with the Sri Lankan sequences indicate animal IDs from Polonnaruwa and Nuwara Eliya, respectively. The Sri Lankan sequences clustered together and formed a sister clade to the common ancestor of the *T. lestoquardi* and *T. annulata* clades.

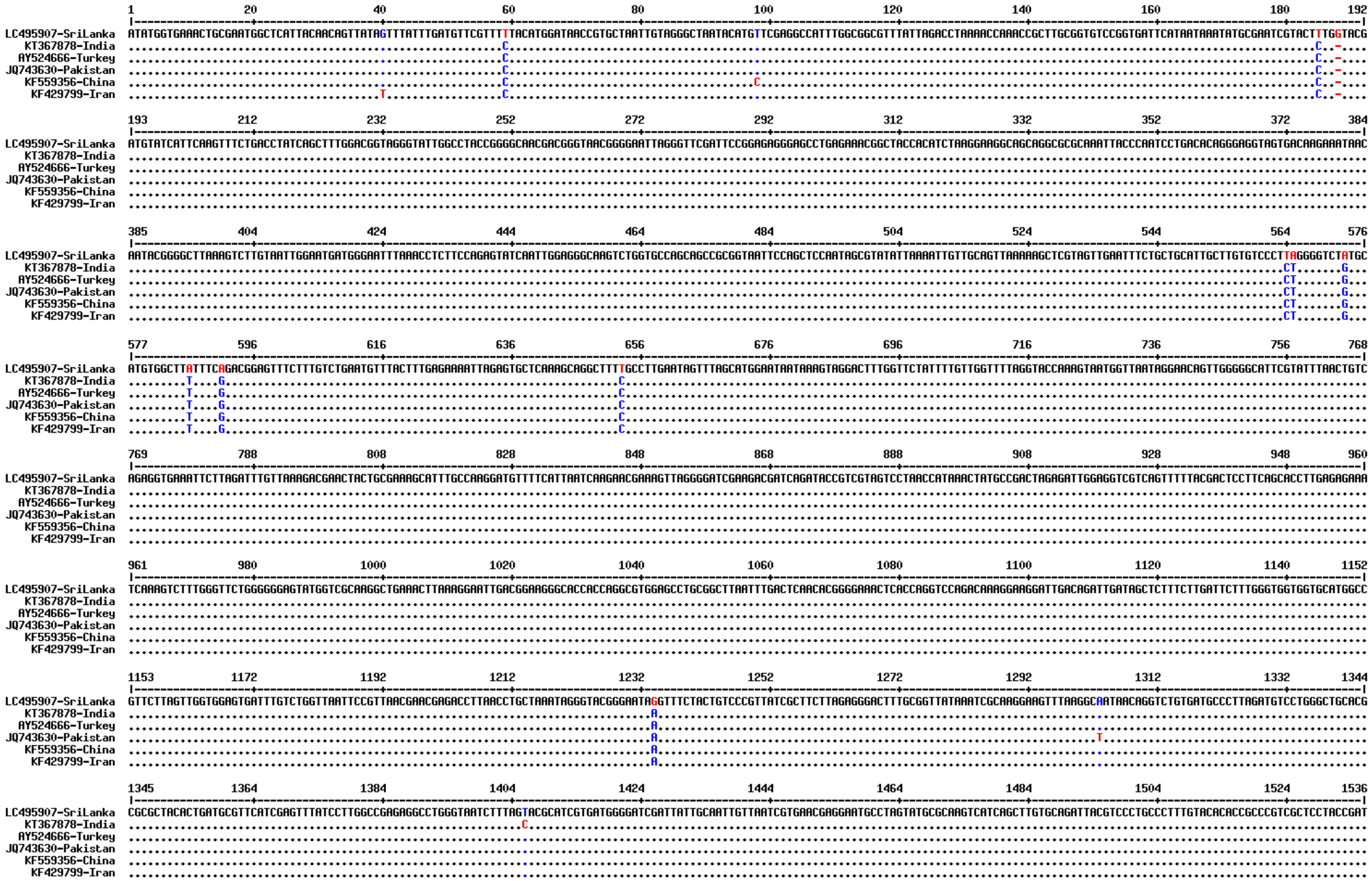

Fig. S7. Multiple alignment of 18S rRNA sequences. A representative 18S rRNA sequence from Sri Lanka was aligned with *T. annulata* 18S rRNA sequences from different countries. Nucleotide bases identical to the reference sequence are indicated by dots, while dashes denote nucleotide deletions. The nucleotides highlighted in red in the Sri Lankan sequence indicate unique SNPs compared with all *T. annulata* sequences.

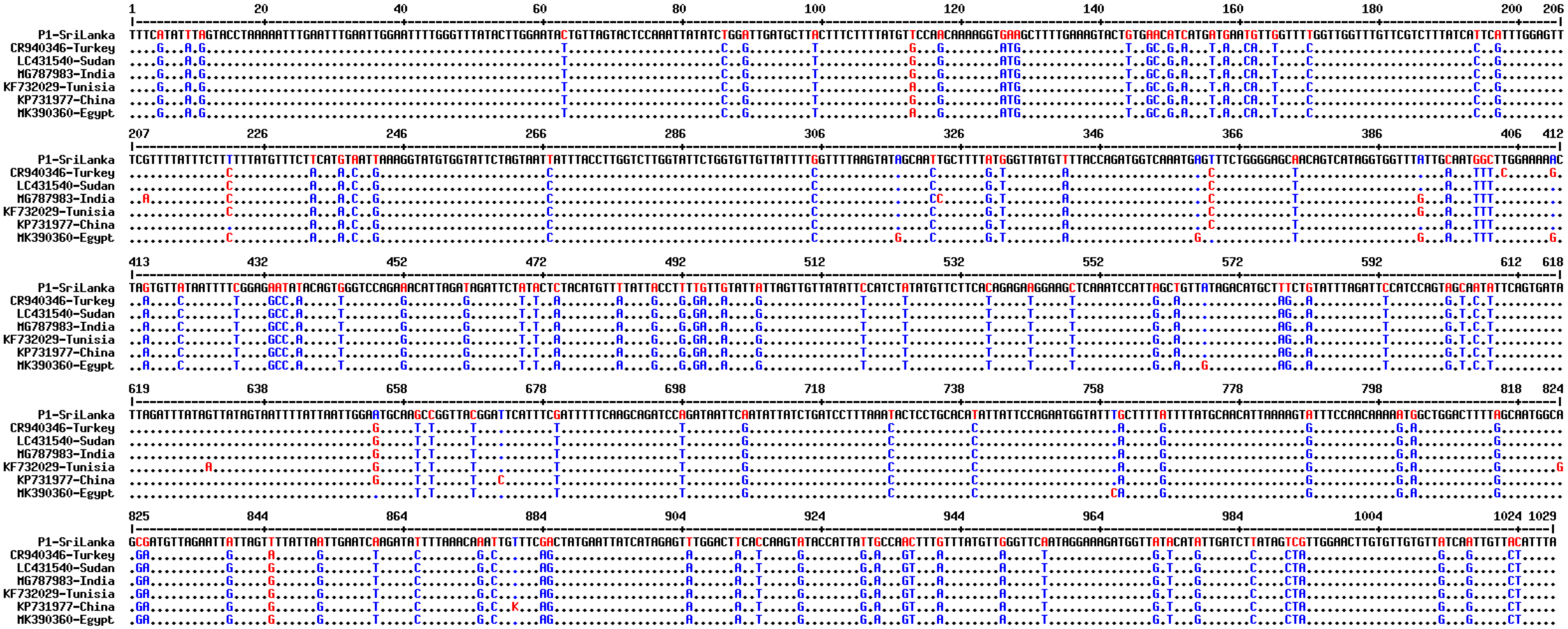

Fig. S8. Multiple alignment of *cob* sequences. A representative *cob* sequence from a Sri Lankan sample was aligned with *T. annulata cob* sequences from different countries. Nucleotide bases identical to the reference sequence are indicated by dots, while dashes denote nucleotide deletions. The nucleotides highlighted in red in the Sri Lankan sequence indicate unique SNPs compared with *T. annulata* sequences. *T. annulata cob* sequences are highly conserved, while the Sri Lankan and *T. annulata* sequences are poorly conserved.

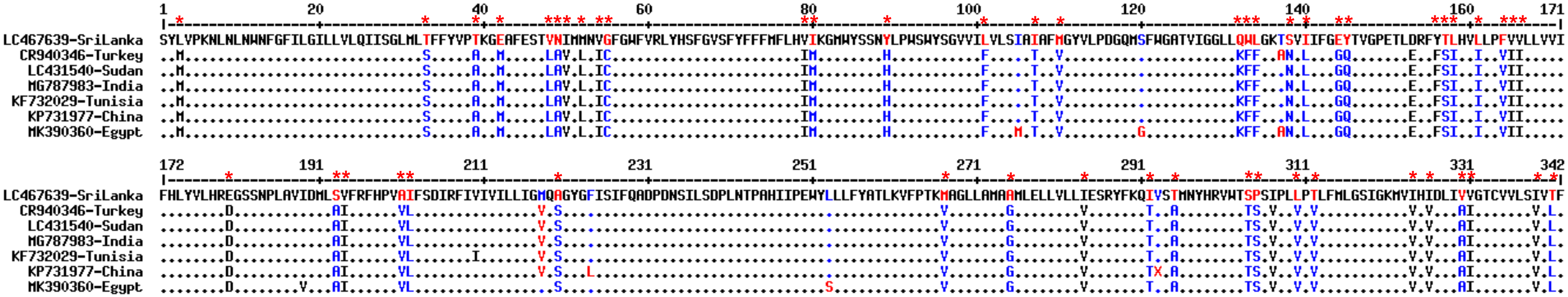

Fig. S9. Multiple alignment of COB amino acid sequences. A representative COB sequence from a Sri Lankan sample was aligned with *T. annulata* COB sequences from different countries. Nucleotide bases identical to the reference sequence are indicated by dots, while dashes denote nucleotide deletions. Asterisks indicate unique amino acid substitutions in Sri Lankan sequences compared with *T. annulata* sequences. *T. annulata* COB sequences are highly conserved, while the Sri Lankan and *T. annulata* sequences are poorly conserved.

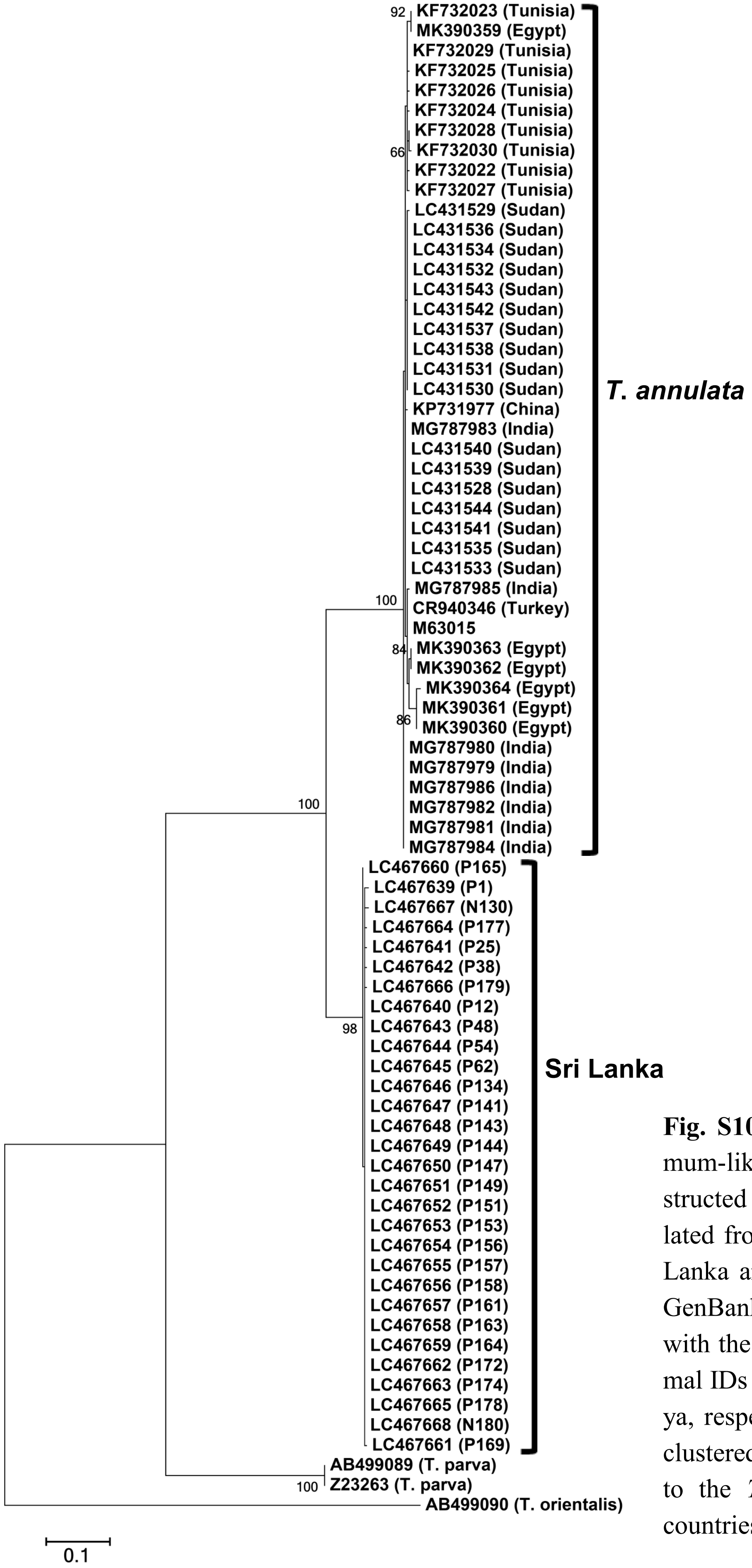

Fig. S10. Phylogeny of the COB. A maximum-likelihood phylogenetic tree was constructed using amino acid sequences translated from 30 *cob* gene sequences from Sri Lanka and the *T. annulata* sequences from GenBank. P and N series numbers provided with the Sri Lankan sequences indicate animal IDs from Polonnaruwa and Nuwara Eliya, respectively. The Sri Lankan sequences clustered together and formed a sister clade to the *T. annulata* sequences from other countries.
